# Creatine transporter (SLC6A8) knockout mice display an increased capacity for *in vitro* creatine biosynthesis in skeletal muscle

**DOI:** 10.3389/fphys.2014.00314

**Published:** 2014-08-26

**Authors:** Aaron P. Russell, Lobna Ghobrial, Craig R. Wright, Séverine Lamon, Erin L. Brown, Michihiro Kon, Matthew R. Skelton, Rodney J. Snow

**Affiliations:** ^1^Centre for Physical Activity and Nutrition, School of Exercise and Nutrition Sciences, Deakin UniversityVIC, Australia; ^2^Division of Neurology, Department of Pediatrics, Cincinnati Children's Research Foundation, University of Cincinnati College of MedicineCincinnati, OH, USA

**Keywords:** energy metabolism, creatine, skeletal muscle, transgenic

## Abstract

The present study aimed to investigate whether skeletal muscle from whole body creatine transporter (CrT; SLC6A8) knockout mice (CrT^-/y^) actually contained creatine (Cr) and if so, whether this Cr could result from an up regulation of muscle Cr biosynthesis. Gastrocnemius muscle from CrT^-/y^ and wild type (CrT^+/y^) mice were analyzed for ATP, Cr, Cr phosphate (CrP), and total Cr (TCr) content. Muscle protein and gene expression of the enzymes responsible for Cr biosynthesis L-arginine:glycine amidotransferase (AGAT) and guanidinoacetate methyltransferase (GAMT) were also determined as were the rates of *in vitro* Cr biosynthesis. CrT^-/y^ mice muscle contained measurable (22.3 ± 4.3 mmol.kg^−1^ dry mass), but markedly reduced (*P* < 0.05) TCr levels compared with CrT^+/y^ mice (125.0 ± 3.3 mmol.kg^−1^ dry mass). AGAT gene and protein expression were higher (~3 fold; *P* < 0.05) in CrT^−/y^ mice muscle, however GAMT gene and protein expression remained unchanged. The *in vitro* rate of Cr biosynthesis was elevated 1.5 fold (*P* < 0.05) in CrT^−/y^ mice muscle. These data clearly demonstrate that in the absence of CrT protein, skeletal muscle has reduced, but not absent, levels of Cr. This presence of Cr may be at least partly due to an up regulation of muscle Cr biosynthesis as evidenced by an increased AGAT protein expression and *in vitro* Cr biosynthesis rates in CrT^−/y^ mice. Of note, the up regulation of Cr biosynthesis in CrT^−/y^ mice muscle was unable to fully restore Cr levels to that found in wild type muscle.

## Introduction

Total creatine (TCr) is the sum of creatine (Cr) and creatine phosphate (CrP). TCr stores in adult rodent and human skeletal muscle are derived predominantly from extracellular Cr uptake via the activity of Na^+^/Cl^−^-dependent creatine transporter (CrT) proteins (Slc6a8) located on the sarcolemmal membrane (Snow and Murphy, 2001). The extracellular Cr is derived from the diet and endogenously synthesized mostly by the kidney and liver (Wyss and Kaddurah-Daouk, 2000). However, a small proportion of adult skeletal muscle TCr stores could be produced from its own Cr biosynthesis given that adult skeletal muscle is known to have low, but detectable *in vitro* activity of both of the enzymes involved in this process (Van Pilsum et al., 1963; Daly, 1985; Cullen et al., 2006). Cr biosynthesis is a two-step reaction process involving the enzymes L-arginine:glycine amidotransferase (AGAT) and guanidinoacetate methyltransferase (GAMT). AGAT produces ornithine and guanidinoacetate (GAA) from arginine and glycine. GAMT catalyses S-adenosyl-L-methionine-dependent methylation of GAA to form Cr and S-adenosyl-L-homocysteine.

Interestingly, McClure et al. (2007) have demonstrated that muscle AGAT and GAMT gene expression and GAMT protein expression are elevated in skeletal muscle from *mdx* mice, a model of human Duchenne muscular dystrophy. This suggests that *mdx* mice may have an enhanced capacity to synthesize Cr in skeletal muscle and that this adaptation may help to maintain muscle Cr levels and limit energy failure in *mdx* skeletal muscle (McClure et al., 2007). Up regulation of AGAT gene and protein activity has also been observed in failing human heart muscle (Cullen et al., 2006). Cullen et al. (2006) hypothesized that elevated AGAT mRNA and enzyme activity in heart failure was an attempt to increase local Cr synthesis to alleviate reductions in cardiomyocyte Cr content typically observed in this disease. In apparent contradiction to the concept that skeletal muscle could up regulate its Cr biosynthesis in circumstances where Cr content was compromised, no Cr was detectable in muscle from a ubiquitous CrT knockout (CrT KO) mouse (Skelton et al., 2011). However, it is possible that the failure to detect Cr in the muscle of these mice was due to the use of a relatively insensitive colorimetric analytical technique.

The aim of the present study was therefore to re-analyse the CrT KO mouse muscle for Cr and CrP content using more specific and sensitive techniques. Furthermore, if Cr was found to be present in CrT KO muscle we aimed to investigate if at least some of this Cr could be produced by an up regulation of muscle Cr biosynthesis. We hypothesized that CrT KO muscle would have low, but detectable levels of Cr and CrP, and that this muscle would display elevated levels of AGAT and GAMT expression resulting in an increased *in vitro* capacity to produce Cr.

## Methods

### Animals

Gastrocnemius muscle samples were obtained from ubiquitous CrT knockout mice (CrT^−/y^) and control mice (CrT^+/y^) bred and raised as described by Skelton et al. (2011). The generation of these mice and tissue extraction was approved by the Cincinnati Children's Research Foundation Institutional Animal Care and Use Committee, protocol #1C06058. The muscle samples were subsequently transported to Deakin University (Australia) on dry ice and stored at −80°C until analysis.

### Metabolite analysis

A portion of each gastrocnemius muscle sample was freeze dried for 24 h, powdered and then weighed. The powdered samples were extracted on ice with 0.5 M perchloric acid and 1 mM ethylenediaminetetraacetic acid and neutralized with 2.1 M potassium hydrogen carbonate. Extracts were analyzed in triplicate for ATP, CrP, and Cr using enzymatic analysis with fluorometric detection as described previously (Harris et al., 1974; Febbraio et al., 1994). TCr was determined by summing the Cr and CrP content. Percent co-efficient of variation for repeated measurement of aqueous standards (200 to 500 μM) for these assays in our laboratory are 2.7, 3.6, and 7.4% for ATP, Cr, and CrP, respectively. The variation of muscle metabolites due to analytical procedure and error employing the same extraction process and assay system is reported to be less than 4% (Harris et al., 1974).

### Gene analysis

RNA was isolated from another portion of the gastrocnemius muscle using TRI-Reagent® Soln. (Ambion Inc., Austin, TX) according to the manufacturer's protocol. RNA concentration was determined by the absorbance at a wavelength of 260 nm using the NanoDrop® ND-1000 spectrophotometer (Thermo Scientific, Rockford, IL). Sample RNA (1.0 μg) was treated with DNAse amplification I (Invitrogen, Carlsbad, CA) before being reverse transcribed to synthesize first strand cDNA using the high-capacity RNA-cDNA reverse transcription kit (Applied Biosystems, Forster, CL). All cDNA was diluted to a working concentration of approximately 5 ng/μl. Efficiency of the RT was determined by Quant-iT™ OliGreen® ssDNA Reagent Kit (Invitrogen).

Real-time quantitative polymerase chain reaction (QPCR) was used to measure mRNA expression as described previously (Wallace et al., 2011). The primers for the CrT gene were designed using the web based software Primer3plus (Untergasser et al., 2007) from the mouse gene sequence (Ensembl Genome Browser). AGAT and GAMT primers were designed against a homologous region across human, mouse, and rat as previously described Ireland et al. (2009). The specific primer sequences are described in Table 1.

**Table 1:** **Sequence of forward and reverse primers for genes of interest**.

**Gene**	**Sense**	**Anti-sense**
CrT	5′ GCC GGC AGC ATG AAT GTC	5′ GTG ATT GTC TTC TAC TGC AAC AC
AGAT	5′ TCA CGC TTC TTT GAG TAC CG	5′ TCA GTC GTC ACG AAC TTT CC
GAMT	5′ TGG CAC ACT CAC CAG TTC A	5′ AAG GCA TAG TAG CGG CAG TC

*CrT, creatine transporter; AGAT, L-arginine:glycine amidinotransferase; GAMT, guanidinoacetate methyltransferase*.

QPCR was performed using a Stratagene Mx3000p QPCR run by MxPro QPCR Software (Stratagene, La Jolla, CA) with SYBR Green PCR Mastermix (Applied Biosystems). Each 20 μl reaction contained 5 μl template and 0.2 μM of each forward and reverse primer. A 3-step QPCR was used to amplify mRNA; initial template denaturing of 95°C for 10 min, followed by 40 cycles of 95°C for 30 s, 60°C for 60 s, and 72°C for 30 s. Fluorescence readings were measured during the last step of cycling and normalized to cDNA content.

### Protein analysis

Western blotting technique was used to measure AGAT and GAMT protein expression in the gastrocnemius muscles of the CrT^−/y^ and CrT^+/y^ mice. GAMT was detected with an affinity purified mouse monoclonal antibody (Monash Antibody Technologies Facility, Melbourne, Australia) made through injection of the antigenic peptide N-terminal aa 125–145. AGAT (also known as GATM) antibody was purchased from Biorbyt Ltd. The antibodies detected a positive band at expected molecular masses (GAMT, 26 kDa; AGAT, 46 kDa) as previously determined (Braissant et al., 2005; McClure et al., 2007).

Muscle protein was extracted using radioimmunoprecipitation assay (RIPA) buffer (Millipore, North Ryde, NSW) containing protease inhibitor cocktail I (Sigma-Aldrich, Sydney, Australia) and Halt® phosphatase inhibitor cocktail (Thermo Scientific, Rockford, IL). Protein concentrations were determined using the bicinchoninic acid (BCA) assay (Pierce Biotechnology, Rockford, USA). Electrophoresis was performed using a 4–12% NuPAGE® Novex Bis-Tris Gel in NuPAGE® SDS MOPS Running Buffer (Invitrogen). Proteins were transferred to a PVDF membrane using a Bjerrum buffer containing 50 mM Tris, 17 mM glycine, and 10% methanol. For GAMT, membranes were blocked with 5% milk powder in PBS for one hour followed by overnight incubation at 4°C with the GAMT primary antibody diluted 1:500 in 5% milk powder in PBS. For AGAT, membranes were blocked with 5% BSA/PBS for 1 h followed by overnight incubation at 4°C with the AGAT primary antibody diluted 1:70 in 5% BSA/PBS. Following washing, all GAMT membranes were incubated for 1 h with rabbit anti-mouse IgG AlexaFluor® 680 (Invitrogen) diluted 1:5000 in PBS containing 50% Odyssey® blocking buffer (LI-COR Biosciences, Lincoln, USA) and 0.01% SDS. AGAT membranes were similarly incubated but with an anti-rabbit secondary antibody. After washing, the proteins were exposed on an Odyssey® Infrared Imaging System (LI-COR Biosciences) and individual protein band optical densities were determined using ImageJ Software (National Institutes of Health, Bethesda, USA). The blots were normalized against the tubulin protein (Sigma-Aldrich).

### *in vitro* creatine biosynthesis

Gastrocnemius muscle from CrT^+/y^ and CrT^−/y^ mice (~20 mg each) were homogenized in potassium phosphate buffer (0.066 M, pH 7.4)(1 mg tissue: 4.5 μl buffer) on ice using an electric homogenizer (Lab-Serv-D-130). The homogenate was subsequently freeze-thawed three times to lyse cell membranes. Rates of Cr synthesis were measured using an adaptation of the methods described (Grazia Alessandri et al., 2004; Ide et al., 2009). One aliquot of the muscle homogenate (33 μl) was incubated in a standard reaction media containing Tris–HCl (5 μl, 100 mM, pH 7.5), arginine (10 μl, 1mM), glycine (5 μl, 1 mM), S-adenosylmethionine (10 μl, 0.25 mM), dithiotheritol (DTT) (5 μl, 2 mM), and 32 μl of potassium phosphate for 2 h at 37°C. After the incubation period the samples were spun twice at 10,000 g, 4°C for 10 min (Eppendorf centrifuge 5402) and the subsequent supernatant filtered (syringe filter 0.2 μm—Phenomenex, USA) and stored on ice prior to Cr analysis. To obtain a reaction blank a second aliquot (33 μl) from each muscle homogenate was added to the reaction media and immediately centrifuged, filtered and stored on ice prior to Cr analysis. A third aliquot (10 μl) from each muscle homogenate sample was analyzed for protein concentration using the BCA Protein Assay Kit (Pierce Biotechnology, Rockford, IL) according to the manufacturer's protocol, and absorbance was measured on a Synergy 2 Microplate Reader (BioTek, Winooski, VT).

Creatine concentration of known Cr standards, the reaction blanks and the two hour samples were measured using high performance liquid chromatography (Agilent 1100 Series system), based on an adaption of the method by Moore et al. (1998). The separation column (i.d. 250 mm × 4.6 mm) was a Luna 10 μm – C18 (2) 100 A (Phenomenex, USA) and the mobile phase was an aqueous solution (pH = 1.5) of 10 mM Na_2_SO_4_, 5 mM H_2_SO_4_, and 10 mM sodium-1-hexane sulfonate (Sigma Aldrich-USA). The mobile phase flow rate was 1 mL.min^−1^ for the first 8 min and then increased to 1.4 mL.min^−1^ for a further 22 min. Elution of Cr was detected by a spectrophotometer (Agilent 1100 G1365B) at a wavelength of 230 nm.

### Statistical analysis

All data are presented as mean ± s.e.m., and were analyzed using a two-tailed Student's two sample *t*-test. Significance was set at *P* ≤ 0.05.

## Results

### Muscle metabolites

Muscle ATP (*P* = 0.002), Cr (*P* = 0.002), CrP (*P* = 0.02), and TCr (*P* = 0.0000014) content were all markedly lower in the CrT^−/y^ mice compared with controls (Table 2). Importantly, Cr and CrP were detected in CrT^−/y^ mice muscle.

**Table 2:** **Gastrocnemius metabolite content in CrT^+/y^ and CrT^−/y^ mice**.

	**CrT^+/y^**	**CrT^−/y^**
ATP	33.7 ± 0.7	15.9 ± 2.4^**^
PCr	36.1 ± 6.9	2.3 ± 0.3^*^
Cr	88.9 ± 8.6	20.1 ± 4.2^**^
Total Cr	125.0 ± 3.3	22.3 ± 4.3^**^

*Values are means ± s.e.m., n = 4 per group. Metabolite content is expressed in mmol.kg^−1^ dry mass. Cr, creatine; PCr, phosphocreatine; TCr, total creatine*,

* *P < 0.05*,

** *P < 0.01*.

### Genes

**Figure 1:**
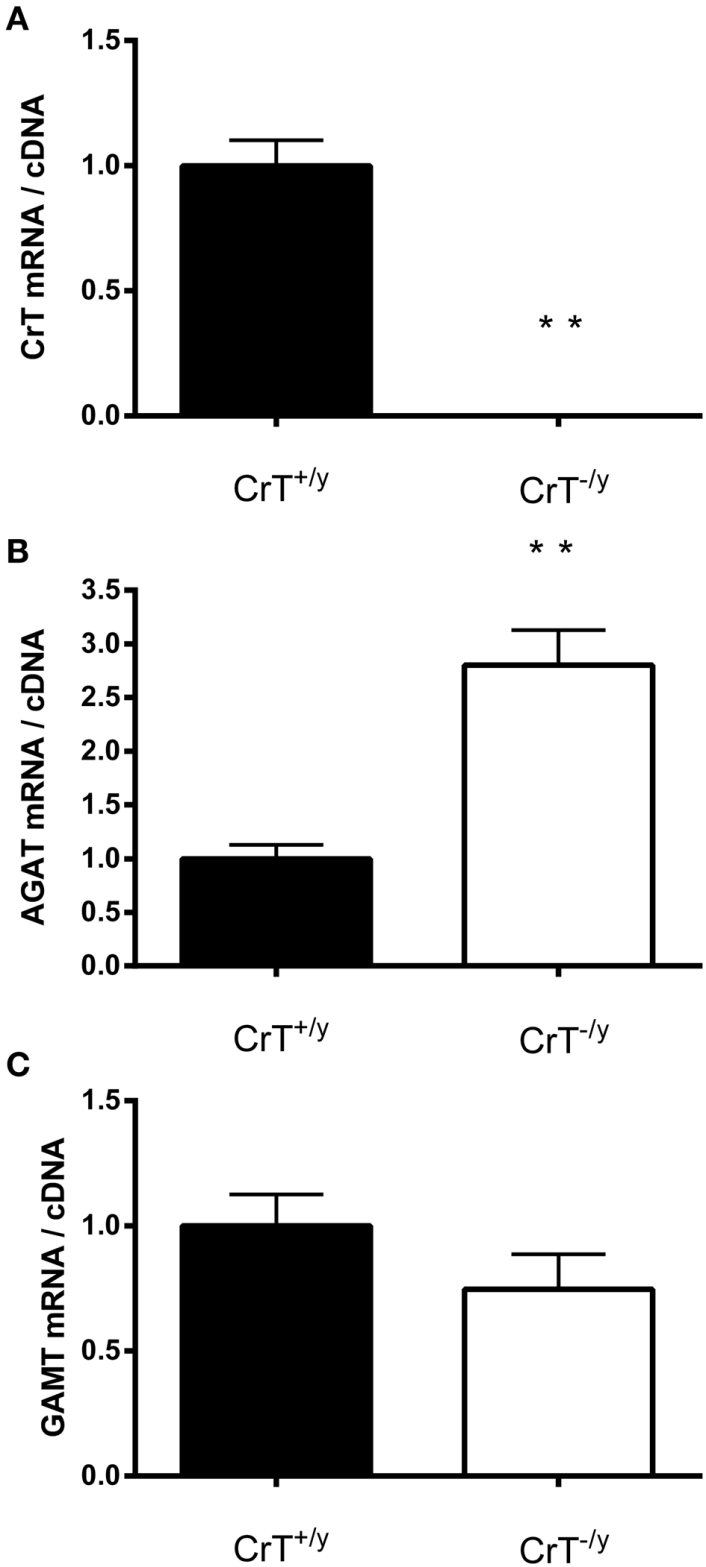
**Gene expression of (A) creatine transporter (CrT) and creatine synthesizing enzymes (B: AGAT and C: GAMT) in CrT^+/y^ and CrT^−/y^ gastrocnemius mice muscle**. Values are means ± s.e.m., *n* = 10 per group. Different from CrT^+/y^^**^P < 0.01.

As expected there was no detectable CrT gene expression in the CrT^−/y^ mice muscle (Figure 1A). Interestingly, there was a different expression response for the two Cr biosynthesis genes AGAT and GAMT. AGAT gene expression was elevated (*P* = 0.00007), but GAMT gene expression was unchanged (*P* = 0.20) in CrT^−/y^ muscle compared with CrT^+/y^ muscle (Figures 1B,C).

### Protein expression and *in vitro* skeletal muscle creatine biosynthesis

**Figure 2:**
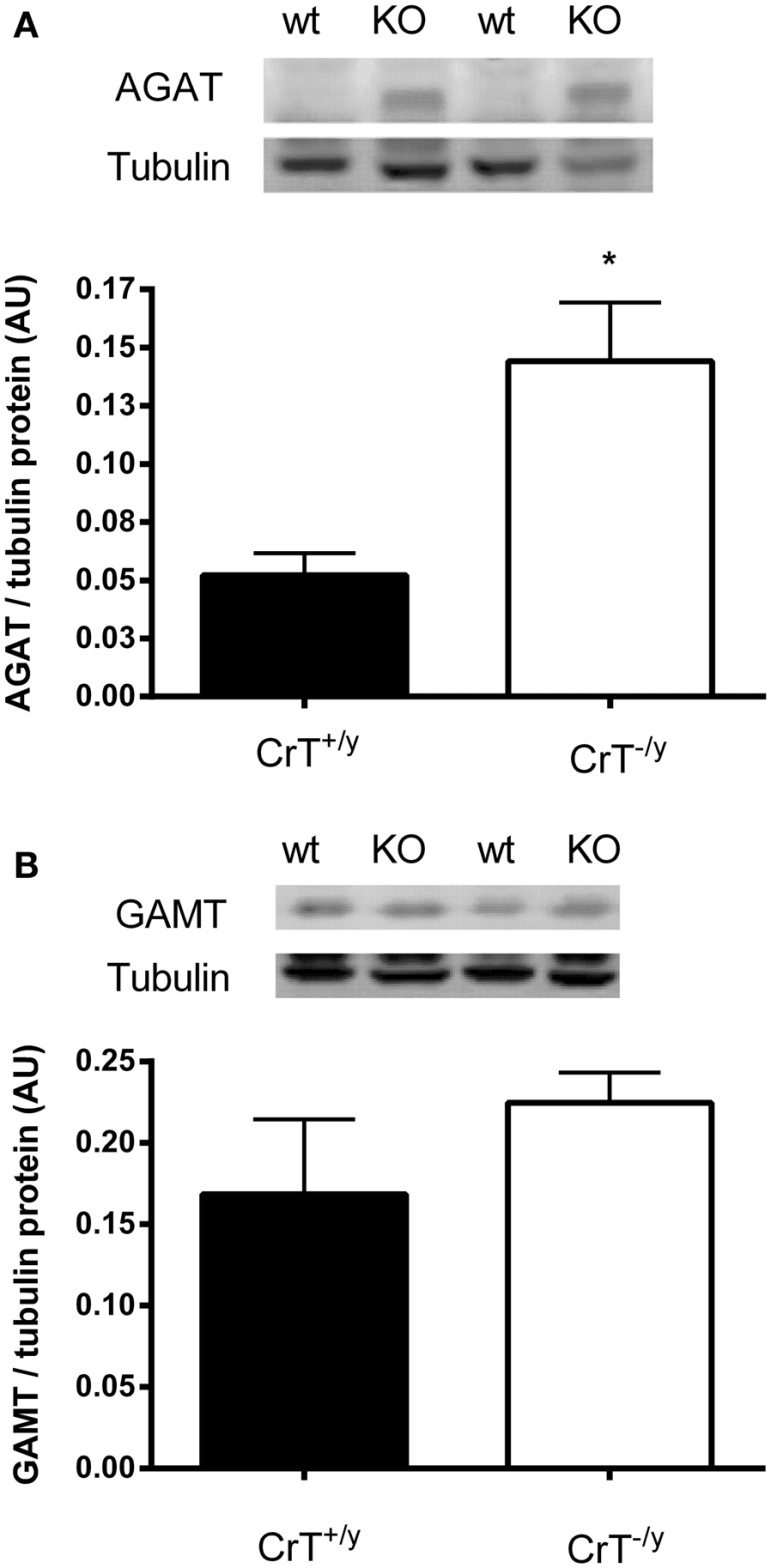
**(A)** AGAT and **(B)** GAMT protein expression in CrT^+/y^ and CrT^−/y^ gastrocnemius mice muscle. Values are means ± s.e.m., *n* = 6 per group. Different from CrT^+/y^^*^P < 0.05.

**Figure 3:**
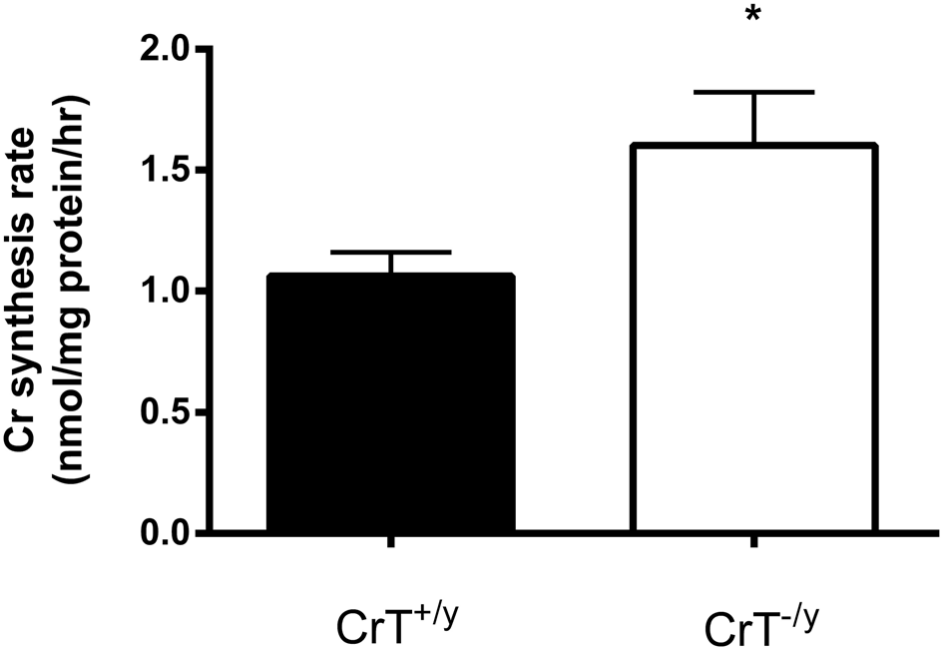
***In vitro* creatine synthesis rates in gastrocnemius muscle of CrT^+/y^ and CrT^−/y^ mice**. Values are means ± s.e.m., *n* = 7 per CrT^−/y^ group and *n* = 10 per CrT^+/y^ group. Different from CrT^+/y^^*^P = 0.05.

The protein expression of AGAT was increased three fold (*P* = 0.01), however GAMT protein was not different (*P* = 0.28) in the CrT^−/y^ mice compared with controls (Figure 2). The *in vitro* Cr biosynthesis rate was 1.5 fold greater (*P* = 0.05) in the CrT^−/y^ mice compared with CrT^+/y^ (Figure 3).

## Discussion

The major findings of the present study were that Cr and CrP were detected in CrT^−/y^ skeletal muscle (e.g., TCr content was 18% of normal muscle) and that *in vitro* Cr biosynthesis rates, as well as AGAT gene and protein expression were elevated in the CrT^−/y^ muscle compared with CrT^+/y^. In contrast to our hypothesis, muscle GAMT gene and protein expression in the CrT^−/y^ were not different to CrT^+/y^ mice. Our findings suggest that at least some of the Cr detected in CrT^−/y^ muscle may be synthesized within the muscle.

The total Cr content determined for CrT^+/y^ gastrocnemius mouse muscle in the present study is similar to that reported previously (Kushmerick et al., 1992), however due to slow sampling and freezing of the muscle the ratio of CrP to Cr we report in Table 2 is not likely to accurately represent that typically found in resting skeletal muscle (Kushmerick et al., 1992). Importantly, we have clearly demonstrated that Cr and PCr can be detected in CrT^−/y^ gastrocnemius muscle in contrast to that reported previously (Skelton et al., 2011). This discrepancy is best explained by the different methods employed to measure these metabolites. In the present study Cr and CrP was analyzed using a widely accepted specific and sensitive enzymatic fluorometric technique on freeze dried muscle (Harris et al., 1974; Febbraio et al., 1994), whereas Skelton et al. (2011) used a less sensitive colorimetric method to analyse wet muscle. The analytical method employed in the present study is likely to be more sensitive. Firstly, because we analyzed freeze dried muscle that is known to concentrate the metabolite of interest by approximately five fold and secondly, because we used a fluorometric detection system as opposed to a spectrophotometric system. Depending upon the analyte, fluorometry is known to have 1000 to 500,000 fold better limits of detection as compared to spectrophotometry.

Of note, the mean gastrocnemius muscle Cr content of wild type mice (CrT^+/y^) reported by Skelton et al. (2011) of 21.82 mmol.kg^−1^ wet muscle (or 94.8 mmol.kg^−1^ dry muscle assuming muscle is 77% water (Hultman and Sahlin, 1980) was very similar to that measured in the present study (88.9 mmol.kg^−1^ dry muscle). The discrepancy between the studies therefore revolves around the muscle Cr content in the gastrocnemius muscle of the CrT^−/y^ mice. The fact that both studies produce similar results when muscle Cr content is high, but not when Cr content is expected to be low lends to support to the contention that Skelton et al's (2011) analysis method using wet muscle tissue was relatively insensitive and therefore unable to detect Cr in CrT^−/y^ mice muscle.

The muscle TCr content measured in this study is expected to be almost exclusively found within the myocytes as extracellular Cr content is calculated to contribute ≤1% of the total Cr measured. This assumes resting muscle extracellular fluid volume is 10% of total muscle water (Hultman and Sahlin, 1980) and serum Cr concentration is 300 μM (Skelton et al., 2011). Of note, the ATP and TCr levels reported for the CrT^−/y^ mouse muscle in the present study are similar to those reported for other models of Cr deficiency such as β-guanidinoproprionic feeding of rats or GAMT-deficient knockout mice (Kan et al., 2005; Williams et al., 2009).

Two interesting questions arise from the presence of Cr in CrT^−/y^ mice muscle. First, via what mechanism does CrT^−/y^ muscle obtain the small but measurable amount of Cr? Secondly, why is this alternative mechanism unable to fully compensate for the deficit in Cr content? Clearly, knockout of the CrT gene should block most, if not all, transport of extracellular Cr into the myofibers. Unfortunately, the present experiment cannot rule out that some Cr enters the muscle via other non-specific transport mechanisms. In support of this possibility, Loike et al. (1988) reported that approximately 10% of total Cr transport into cultured rat L6 muscle cells could occur via a sodium independent mechanism and therefore, at least in this muscle cell culture system, all sarcolemmal Cr transport could not be attributed to CrT activity alone. Further research using radiolabeled Cr is required to determine the actual existence and extent of non-CrT dependent Cr uptake into skeletal muscle of the CrT^−/y^ mice.

In addition to, or alternatively, skeletal muscle may up regulate its own Cr biosynthesis when myocyte Cr content is compromised (Cullen et al., 2006; McClure et al., 2007). The elevated rates of *in vitro* Cr biosynthesis coupled with increased AGAT gene and protein expression in the CrT^−/y^ mouse muscle strongly indicates that skeletal muscle Cr synthesis is switched on to a greater extent and may be responsible for at least some of the muscle Cr content in this model. This contention is supported by the fact that AGAT is considered to be the rate limiting enzyme of Cr biosynthesis (Walker, 1979). Therefore any increase in GAA production by AGAT should lead to Cr production in skeletal muscle, provided GAMT is present in this tissue, which we have clearly demonstrated to be the case (see Figure 2). The finding that AGAT gene and protein expression can be up regulated in tissues not normally expected to rely on its own Cr production is consistent with that reported for the failing human heart muscle and *mdx* mouse skeletal muscle (Cullen et al., 2006; McClure et al., 2007).

Interestingly, GAMT gene and protein were expressed to a similar extent in both wild type and CrT^−/y^ muscle contrary to our hypothesis and in apparent contrast to that reported by McClure et al. (2007) when studying *mdx* mouse muscle. Based on previous research we expected that GAMT gene and protein expression would be up regulated in CrT^−/y^ mice because the observed disturbance in high energy phosphagen content (see Table 2) should activate AMP kinase (Williams et al., 2009). AMP kinase is known to activate the p53 transcription factor, which is able to increase GAMT gene transcription (Ide et al., 2009). It should be noted that Cr levels do not directly regulate GAMT gene expression, at least in liver cells (Wyss and Kaddurah-Daouk, 2000).

It is unclear why GAMT gene and protein expression in the CrT^−/y^ muscle was not up regulated as hypothesized. Nevertheless this did not prevent an increase in Cr biosynthesis capacity when muscle homogenates were incubated with arginine, glycine, and S-adenosylmethionine, suggesting that AGAT, but not GAMT activity, was rate limiting in the *in vitro* assay system employed in the present study. The Cr synthesis rate we observed in CrT^+/y^ mice gastrocnemius muscle (1.06 ± 0.07 nmol Cr/mg protein/h) was slightly higher than reported for rat quadriceps muscle (Daly, 1985) GAMT activity (0.39 and 0.82 nmol Cr/mg protein/h), but markedly lower than that reported for rat liver (Wyss and Kaddurah-Daouk, 2000) GAMT activity (12-20 nmol Cr/mg protein/h).

Even though CrT^−/y^ muscle displayed increases in AGAT protein expression and *in vitro* Cr synthesis capacity, there still remained a marked deficit (~80%) in muscle Cr content in these mice when compared with CrT^+/y^ mice. This observation indicates that the elevation in endogenous muscle Cr synthesis capacity and/or non-CrT dependent muscle Cr uptake capacity is insufficient to overcome the loss of Cr transport across the sarcolemma associated with the absence of CrT protein expression. In the case of insufficient Cr synthesis capacity it remains unclear why this occurs, but may be related to insufficient intramuscular substrate supply to activate AGAT and/or GAMT to high enough rates, endogenous inhibition of one or both these enzymes and/or an elevated Cr leakage rate from the muscle counteracting against any increase in endogenous Cr production rates.

Clearly the functional significance of CrT gene knockout on skeletal muscle was not investigated in the present study. We observed that gastrocnemius mass was considerably smaller in CrT^−/y^ mice compared with CrT^+/y^ mice suggesting that muscle morphology was affected. Although speculative, TCr depletion in the CrT^−/y^ mice muscle would probably trigger similar adaptations to that observed in other models of chronic muscle Cr depletion such as β-guanidinoproprionic acid feeding and AGAT or GAMT gene knockout models (Kan et al., 2005; Nabuurs et al., 2013; Oudman et al., 2013). For example chronic muscle Cr depletion has typically resulted in ATP depletion, muscle fiber atrophy, increased mitochondrial content, altered contractile performance and a shift toward a greater proportion of slow twitch fibers.

In conclusion, the present experiment clearly demonstrated that Cr was present in CrT^−/y^ mice gastrocnemius muscle. At least some of this Cr may result from an increased muscle capacity to synthesize Cr, however non-specific Cr transport across the sarcolemmal cannot be excluded as a possible contributor to the Cr content observed in CrT^−/y^ mice muscle.

## Author contributions

All authors were either involved conception and design of the work (Aaron P. Russell, Rodney J. Snow, Matthew R. Skelton) or the acquisition, analysis or interpretation of the data (Séverine Lamon, Aaron P. Russell, Lobna Ghobrial, Craig R. Wright, Erin L. Brown, Michihiro Kon). All authors were involved in critically evaluating drafts of the manuscript and provided final approval of the submitted version to be published. All authors have agreed to be accountable for all aspects of the work and will ensure that questions related to accuracy and integrity will be appropriately investigated.

### Conflict of interest statement

The authors declare that the research was conducted in the absence of any commercial or financial relationships that could be construed as a potential conflict of interest.
